# Physicochemical properties and sensory quality of Motlopi (*Boscia albitrunca*) coffee prepared using different temperature-time combinations

**DOI:** 10.1016/j.heliyon.2022.e10829

**Published:** 2022-10-01

**Authors:** Kenanao Otsogile, Eyassu Seifu, Geremew Bultosa

**Affiliations:** Department of Food Science and Technology, Botswana University of Agriculture and Natural Resources, Private Bag 0027, Gaborone, Botswana

**Keywords:** *Boscia albitrunca* roots, Physicochemical properties, Motlopi coffee, Roasting temperature, Roasting time, Sensory quality

## Abstract

Motlopi coffee is a beverage prepared from roots of an indigenous tree *Boscia albitrunca* and consumed in Botswana. To date, there is no published report about the quality characteristics of Motlopi coffee. This study was conducted to develop an improved Motlopi coffee by varying roasting time and temperature and assess its physicochemical properties and sensory quality. The roasting temperatures considered were 150 °C and 160 °C with roasting time of 10, 15 and 20 min at each temperature. The study showed that bulk density of ground coffee was significantly (p < 0.05) decreased at 160 °C than at 150 °C and pH of Motlopi coffee was generally higher at 150 °C than at 160 °C. The total soluble solids (TSS) of Motlopi coffee decreased with increasing roasting time at 150 °C; however, it increased with increase in roasting time at 160 °C. Browning index (BI) tended to increase with increase in roasting time both at 150 and 160 °C and was significantly (p < 0.05) higher at 160 °C than at 150 °C. The titratable acidity (TA) decreased with an increase in the roasting time both at 150 and 160 °C and was significantly (p < 0.05) higher at 150 °C than at 160 °C. The traditionally prepared Motlopi coffee had comparable TA with treatment 2 (150 °C for 15 min). The consumer acceptability test showed that the laboratory made Motlopi coffee had significantly higher (p < 0.05) scores for taste, body and overall acceptability than the traditional Motlopi coffee. Roasting Motlopi roots at 150 °C for 15 min resulted in Motlopi coffee of good physicochemical properties and sensory quality.

## 1 Introduction

*Boscia albitrunca* (the shepherd’s tree) is usually found in the drier parts of southern Africa where it is often referred to as the Tree of Life as it offers sustenance to both humans and animals. It is a small to medium-sized tree reaching heights of 7 m and has an attractive dense, round to spreading crown. The trunk is distinctly smooth and white or whitish grey with bare stems (Palmer and Pitman, 1972; Palgrave, 2002).

*B. albitrunca* is regarded as a multipurpose tree in southern Africa where it is used as herbal medicine for treatment of variety of animal and human diseases (Maroyi, 2019). In Namibia, fresh leaf and root extracts of *B. albitrunca* are used to treat lung and liver infections in cattle and goats whereas leaf and bark extracts are used to manage syphilis in humans (Chinsembu, 2015). In Mozambique, the leaves of *B. albitrunca* are used to treat diarrhoea, haemorrhoids, muscular pain and constipation (Chinsembu, 2015). Bapedi traditional healers in South Africa use roots of *B. albitrunca* to treat HIV/AIDS-related symptoms (Chinsembu, 2015). It was also reported that the green fruits of *B. albitrunca* are used to treat epilepsy while the leaf extracts are used to treat eye related aliments (Pendota et al., 2015).

In Botswana, *B. albitrunca* is known as Motlopi tree. It is consumed by livestock and wild animals and used for different purposes by the local people. The roots, when dried, roasted and ground are used as a substitute for coffee or can be pounded into a white meal to make porridge (Maroyi, 2019; Leistner, 2000). The Bushmen use *B. albitrunca* trunks to catch rainwater and its berry-like flower buds are edible (Leistner, 2000). A concoction made from the leaves is used to treat eye inflammation in cattle and haemorrhoids for humans (Maroyi, 2019). It provides shade during hot sunny days. *Boscia albitrunca* is well known for its coffee brewed from the roots (Maroyi, 2019).

*Boscia albitrunca* is useful in the brewing of a hot delicious coffee like beverage called Motlopi coffee. Traditionally, Motlopi coffee is prepared by cleaning the root of Motlopi tree and shredding it into smaller pieces using a knife. The tiny pieces are then pounded into a pulp using a mortar and pestle. The pulp is allowed to dry in the sun and dried pulp is roasted in a pot until it is brown. During roasting a little bit of oil is used to prevent the roots from sticking to the pan. Brown sugar is then added to the pot and the contents of the pot are mixed until they start to shine because of the heat. The contents are then emptied onto a flat surface and allowed to dry (Lotter, 2002). The mixture is then ground into a powder using a grinding stone. It is then sifted and is ready to be prepared just like an ordinary coffee.

In addition to its use as coffee substitute, the root of *B. albitrunca* is used to preserve and add flavour to fermented milk in Botswana and Namibia (Maroyi, 2019). Bille (2009) reported that *B. albitrunca* root is used in the processing of the Namibian traditional fermented buttermilk called Omashikwa. He indicated that *B. albitrunca* root increases viscosity, reduces syneresis and inhibits bacterial growth in Omashikwa.

The quality of coffee is determined by different factors of which processing condition plays an important role in influencing the aroma and taste of coffee. The quality attributes of coffee such as taste, aroma and colour are greatly influenced by processing conditions especially roasting time and roasting temperature (Pinheiro et al., 2021).

Roasting is a key step in coffee processing, and it changes the chemical, physical, structural and sensory properties of green beans through heat-induced reactions (Pereira et al., 2021). The characteristic aroma of coffee is influenced by the volatile compounds produced during the roasting process (Pereira et al., 2021). Similarly, the typical coffee flavour is generated during the roasting process (Pereira et al., 2021). Roasting involves chemical changes that are induced in the green coffee beans with concomitant change in the physical structure of the coffee and these changes depend on the time and temperature applied during the roasting process (Pereira et al., 2021).

Process conditions in particular the time-temperature combination used during roasting significantly influence the physical and chemical properties of roasted coffee as a function of heat transfer (Pereira et al., 2021). Physical and chemical transformations of the green coffee occur as a result of the thermal treatment (roasting), which include change in colour, texture, density, and size (Pereira et al., 2021). Variations in time-temperature during roasting are the major factors that determine the distinct aroma compounds produced in the coffee. Thus, careful control of roasting time and temperature is required to reach a specific flavour profile (Pereira et al., 2021).

To date, there is no documented information that reports quality characteristics of the traditionally prepared Motlopi coffee, and no attempt has been made to improve the production process and quality of Motlopi coffee. This work was conducted to improve the production process of Motlopi coffee and assess its quality characteristics. The results of the present work provide important baseline information that could be used for further improvements of the quality of Motlopi coffee in the future.

The objective of this study was to develop Motlopi coffee from *Boscia albitrunca* roots by varying roasting time and temperature and assess its physicochemical properties and sensory quality.

## 2 Materials and methods

### 2.1 Description of the study area

The study was conducted in Gaborone city, which is situated between Kgale and Oodi Hills, on the Notwane River in the southern east corner of Botswana and 15 km from the South African boarder. The city lies at an elevation of 1010 m above sea level. The climate of Sebele where the experiment was conducted is semi-arid.

The winter from May to August is dry and sunny, mild during the day but cold at night especially in the south. From October to March, the average temperature is 30 °C or slightly higher. The moisture in the atmosphere is high in the months of December to March which is the rainy season. In winter from May to August, the sun shines with no rain, daytime temperatures are pleasant while the nights are cold. In Gaborone, January is the hottest month with an average temperature ranging from 37°C to 40 °C and the coolest month is July at 13.15 °C with the most daily sunshine hours at 11 am in December.

### 2.2 Collection of Motlopi roots

About 2 kg of Motlopi roots were collected from Motlopi tree in Maunatlala village at Tswapong region of Botswana in April 2021. The roots were cut using an axe and enough roots were harvested to produce the coffee. Medium sized roots were preferred as they are rich in flavor and easy to cut.

### 2.3 Preparation of Motlopi coffee

#### 2.3.1 Traditional process

**Figure 1:**
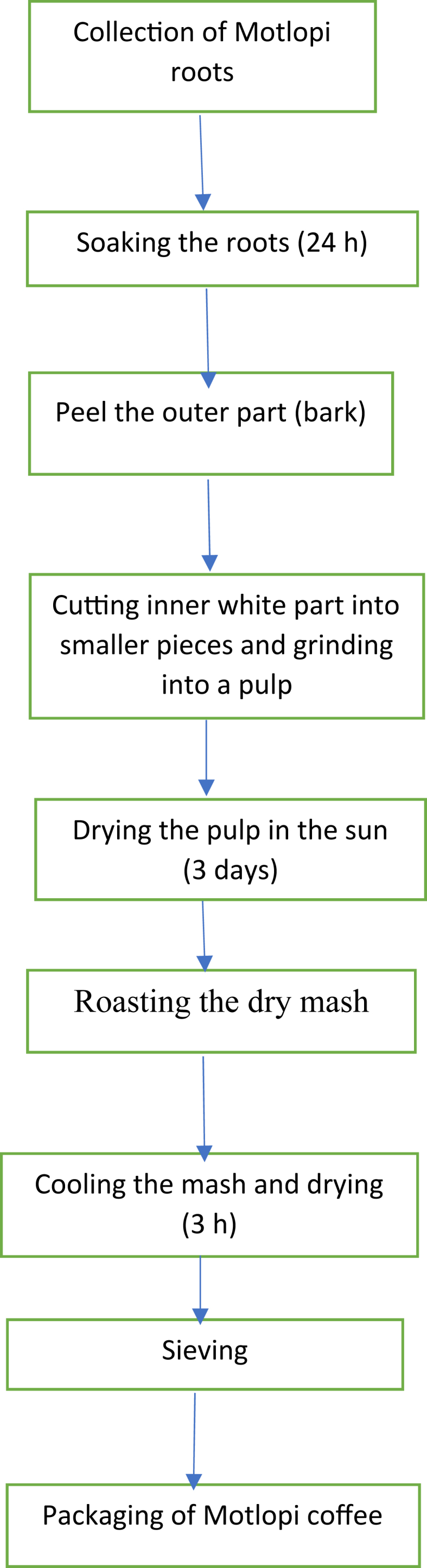
Flow diagram of the traditional Motlopi coffee preparation method.

In the traditional process, roots are cut out from Motlopi tree. The roots are cleaned to remove physical contaminants such as soil, pests, and weeds. The roots are then immersed into a bowl full of clean tap water. The roots are left covered in water for three days to soak and soften. After three days the outer part of the bark is peeled off. The white inner part of the roots is cut into finer particles and ground into a pulp using a mortar and pestle. The pulp is allowed to dry in the sun for three days and the dried pulp was roasted using a bit of lard so that it does not stick to the pan and to enhance the softness. The powder is sieved and packaged. In current study, Motlopi coffee powder prepared in the traditional way was bought from a local vender at Main Mall in Gaborone city. The steps in the preparation of the traditional Motlopi coffee are indicated in Figure 1.

#### 2.3.2 Improved process

Motlopi coffee was prepared in the laboratory under controlled conditions by varying the temperature and time of roasting of the Motlopi pulp as indicated in Table 1. About 1 cm cube of solid vegetable oil (Holsum Fat, Sime Darby Oils Pty Ltd, Boksburg, South Africa) was cut and rubbed on the surface of the pan before roasting to prevent sticking of the roots to the pan (Pujadas Sauté Pan, 4.7 L capacity, 32 cm diameter, stainless steel material). All the other steps of the preparation of Motlopi coffee were the same as for the traditional method except the roasting temperature and roasting time. The roasting temperature and time were chosen after a preliminary experiment in the laboratory. Very high temperature resulted in a charred Motlopi coffee while very short time resulted in under roasted Motlopi coffee.

**Table 1:** Experimental design including process factors (roasting temperature and time) and their levels.

Treatments	Temperature (ᵒC)	Time (min)
1	150	10
2	150	15
3	150	20
4	160	10
5	160	15
6	160	20

### 2.4 Preparation of the brewed coffee for sensory evaluation

The Motlopi coffee was prepared in a Turkish coffee style where finely ground coffee powder was mixed with hot water for making coffee without filtering. This is the way how people drink Motlopi coffee in Botswana. About 240 mL of water was boiled in a kettle. After the water has boiled, it was poured into a cup (250 mL). Then about 12 g of Motlopi coffee powder was added into the cup containing boiling water. The content was stirred with a spoon and the coffee was allowed to stand for a minute to dissolve the brew and then was made ready to drink.

### 2.5 Experimental design

A full factorial design involving two factors: roasting temperature (2-levels) and roasting time (3-levels) was used as the experimental design as shown in Table 1. The Motlopi coffee was prepared by varying the process factors, that is, roasting temperature and roasting time. Two roasting temperatures 150 and 160 °C and three roasting times 10, 15 and 20 min were used. A total of 6 treatments were applied and the quality characteristics of the experimental coffees was compared with the quality of Motlopi coffee prepared in the traditional way which was used as a control. The experiment was replicated three times.

### 2.6 Physiochemical properties

#### 2.6.1 Titratable acidity

Titratable acidity of Motlopi coffee was determined by measuring 5 g of the powder and mixing it with 20 mL of distilled water to make a solution. The solution of coffee powder and distilled water was filtered using a funnel and coffee filter paper called chemex coffee filters (Chemex ® Corp., Pittsfield, Massachusetts, USA). The solution (10 mL) was transferred into a beaker. Three drops of phenolphthalein were added as an indicator and the solution was titrated with 0.1 N NaOH (Rao et al., 2020). The titratable acidity was calculated using the formula (Eq. (1)):

(1) %Acid = [(N x V x Eqwt)/(V∗10)]

where:

N = normality of standard NaOH solution used for titration,
V = volume of standard NaOH used for titration in mL consumed on the titration,
Eqwt = equivalent weight of the dominant acid (chlorogenic acid) (formula weight of chlorogenic acid divided by the number of hydrogen ions in the acid molecule that was titrated)
V = sample size in milliliters.

#### 2.6.2 pH

The pH of Motlopi coffee was measured by mixing 3 g of Motlopi powder with 20 mL of distilled water and pH of the solution was measured using a glass electrode attached to the pH meter (Orion Star A111 pH Meter, Thermo Fisher Scientific Inc, USA) after calibration of the glass electrode with buffer pH 4, 7 and 9 (Clifford, 1985b).

#### 2.6.3 Total soluble solids

Total soluble solids (TSS) of the powdered coffee samples were determined by mixing coffee powder (2 g) with distilled water (10 mL) to make a solution. The solution was filtered using chemex coffee filter paper (Chemex ® Corp., Pittsfield, Massachusetts, USA). Two drops of the filtrate were placed on a refractometer (Model A21341-CC J-257, Rudolph Research Analytical, Hackettstown, USA) using a dropper after which the readings were taken and recorded (Nguyen et al., 2019). The total soluble solids were calculated with the following formula (Eq. (2)):

(2) %TSS= 0.85 x %Brix

where:

%Brix = reading taken from the refractometer

#### 2.6.4 Density of the powder

The bulk density (Pb) of the powder was measured by pouring the ground coffee sample into a graduated measuring cylinder of defined volume (50 mL). The volume was read and recoded (Vb). The sample was then placed in a weighing boat and mass was taken on analytical balance from the volume and mass measured. The bulk density was calculated using the following equation (Eq. (3)) (Fikry et al., 2019).

(3) Pb=(m2−m1)/Vb

where m1 = mass of weighing boat, m2 = mass of weighing boat + mass of coffee powder and Vb = volume occupied by the powder.

#### 2.6.5 Browning index

The browning index (BI) of the coffee brew was measured after coffee brew (50 μL) was diluted up to 2 mL with demineralized water. The browning index was measured by reading the absorbance of samples at 420 nm, after exactly 2 min in a 3-mL capacity cuvette (1 cm length) using a visible spectrophotometer (Jenway 6300 Spectrophotometer, Cole-Parmer Ltd, Staffordshire, UK) (Cämmerer and Kroh, 2006). The BI was calculated using the following formula (Eq. (4)):

(4) BI = X-0.31 × 100/0.172

X = Chromaticity coordinate calculated from XYZ values.

### 2.7 Sensory analysis

Sensory acceptability test was conducted on brewed Motlopi coffee samples. The experimental coffee (which was prepared by roasting Motlopi roots at 150 °C for 15 min) and traditionally made Motlopi coffee samples (three-digit coded samples) were served in random order to 30 untrained panelists to evaluate how much they like the sensory qualities (aroma, colour, body, taste, and overall acceptability) using a 9-point hedonic scale (where 1 = dislike extremely; 2 = dislike very much; 3 = dislike moderately; 4 = dislike slightly; 5 = neither dislike nor like; 6 = like slightly, 7 = like moderately; 8 = like very much and 9 = like extremely). After and in between evaluation of each sample, the panelists rinsed their mouth with water to avoid carry over effect (Chung et al., 2013). The panelists were informed about the objectives of the study prior to the start of the experiment and they gave their informed consent to participate in the sensory evaluation of the product.

### 2.8 Statistical analysis

Data on the physicochemical parameters was subjected to a one-way analysis of variance (ANOVA) and the treatment means were separated using the least significant difference (LSD) at alpha = 5% using the Statistical Analysis System (SAS) software. Two-way ANOVA was used for analysis of the sensory score data by including panelists as a block and LSD was used for mean separation.

## 3 Results and discussion

### 3.1 Physicochemical properties

The physicochemical properties of Motlopi coffee are indicated in Table 2. A decrease in the pH was observed with a prolonged duration of roasting time at 150 °C (Table 2). At 160 °C, the pH of Motlopi coffee decreased at a roasting time of 15 min but remained the same at 10- and 20-min roasting times (Table 2). When comparing the two roasting temperatures, the pH of Motlopi coffee is generally higher at 150 °C than at 160 °C (Table 2). The traditionally prepared Motlopi coffee had the lowest pH whereas the highest pH value was recorded for treatment 1 (150 °C for 10 min) (Table 2). According to Nebesny and Budryn (2006), the higher the roasting temperature, the least acidic the coffee becomes. Sunarharum et al. (2014) reported that coffee contains several acids that are useful for developing the distinctive aroma and taste of coffee. The presence of these acids affects the degree of coffee acidity. Coffee with high acidity indicates that the coffee has good aroma and taste due to the existence of volatile acidic compounds (forming aroma) and flavor forming acids. However, these in both *Coffea arabica* and *Coffea canephora* vary with type of acids as different organic acids impart different sensory taste attributes such as sour, bitter, astringent, caramel and some are noted as enhancer of fruit flavors (Yeager et al., 2021). Roasted coffee quality was indicated to be negatively associated with acetic acid and overall coffee acidity (Yeager et al., 2021). Venkatachalam and Sengottian (2016) explains that temperature also has an effect on both pH buffers and solutions, as the hydrogen ion activity will increase with increasing temperature.

**Table 2:** Physicochemical properties of Motlopi coffee prepared using different temperature time combinations in comparison with traditional Motlopi coffee.

Temperature (°C)	Time (minutes)	pH	Density (kg/cm³)	Total soluble solids (mg/L)	Browning Index (%)	Titratable acidity (%)
150	10 (T1)	4.95^a^ ± 0.01	0.39^a^ ± 0.07	1.70^c^ ± 0.04	17.35^e^ ± 9.38	7.49^a^ ± 0.09
	15 (T2)	4.64^b^ ± 0.02	0.39^a^ ± 0.04	1.25^d^ ± 0.03	74.33^d^ ± 9.39	5.59^b^ ± 0.08
	20 (T3)	4.54d^e^ ± 0.03	0.37^b^ ± 0.05	0.96^e^ ± 0.02	73.00^d^ ± 9.37	5.08^c^ ± 0.06
160	10 (T4)	4.57^cd^ ± 0.04	0.32^c^ ± 0.01	1.31^d^ ± 0.01	251.33^c^ ± 9.28	4.64^d^ ± 0.05
	15 (T5)	4.54^e^ ± 0.06	0.34^c^ ± 0.03	1.67^c^ ± 0.05	420.00^b^ ± 9.48	3.95^e^ ± 0.07
	20 (T6)	4.57^c^ ± 0.05	0.34^c^ ± 0.02	2.47^a^ ± 0.07	451.00^a^ ± 9.38	3.69^e^ ± 0.04
	Control	4.53^c^ ± 0.08	0.37^c^ ± 0.07	1.98^b^ ± 0.06	415.00^b^ ± 9.49	5.57^b^ ± 0.08

T1 = treatment 1 at 150 °C for 10 min; T2 = treatment 2 at 150 °C for 15 min; T3 = treatment 3 at 150 °C for 20 min; T4 = treatment 4 at 160 °C for 10 min; T5 = treatment 5 at 160 °C for 15 min; T6 = treatment 6 at 160 °C for 20 min; Control = traditional Motlopi coffee; Means with different superscript letters in a column are significantly different (p < 0.05); Values in the Table are averages of triplicate observations.

The density of the ground coffee powder increased with increasing roasting time at both roasting temperatures of 150 and 160 °C (Table 2). The density was lower at 160 °C than at 150 °C (Table 2). The lowest density was recorded for treatment 4 (160 °C for 10 min) and the highest density was recorded for treatments 1 and 2 (150 °C for 10 min/150 °C for 15 min) (Table 2). According to Somporn et al. (2011), bulk density is a property of powder and granules especially used in reference to mineral components, chemical substance and ingredients. These authors further explain that density changes with temperature. As substances are heated, the volume increases because the faster moving molecules are further apart. Getaneh et al. (2020) reported that the bulk density of coffee decreased as the roasting time and temperature increase. The authors explain that the root cause of the decrease of density could be attributed to the rise of the volume. This could be due to the increase of porosity of the root structure, as determined by the rise in pressure of the internal gases (released CO_2_, water, and volatile substances) resulting from pyrolysis reactions. Similarly, Dalia et al. (2014) reported that the increase in coffee bean volume resulted from the softening of the cellulose bean structure coupled to the increase in pressure from the release of pyrolysis products. The authors further explained that the bulk density reduction is attributed to increase in volume and internal gas formation of characteristic porous structure in the roasted coffee bean.

The total soluble solids (TSS) of Motlopi coffee decreased with increasing roasting time at 150 °C; however, it increased with roasting time at 160 °C (Table 2). The highest TSS was recorded for treatment 6 (160 °C for 20 min) and the lowest TSS was recoded for treatment 3 (150 °C for 20 min) (Table 2). According to Youn and Chung (2012), coffee shall exhibit a brew strength measured in total dissolved solids of 11.5–13.5g which corresponds to 1.15–1.35% on the brewing control chart. It was found that the higher the roasting temperature, the higher total soluble solids content of coffee. Roasting at a higher temperature and for longer time causes increased evaporation of water from the product. Increased evaporation results in reduction in the water content and a concomitant increase in the percentage of TSS. Mursalin et al. (2019) reported that evaporation of water during heating caused a decrease in water content but an increase in the solids content of coffee. Significantly higher TSS were also reported by Cordoba et al. (2021) for hot brewed coffee roasted at higher temperature as compared to coffee roasted at lower temperature. They attributed the high TSS in coffee roasted at higher temperature to the effect of high temperature on the microstructural components of the coffee beans which make compounds more readily extractable.

**Figure 2:**
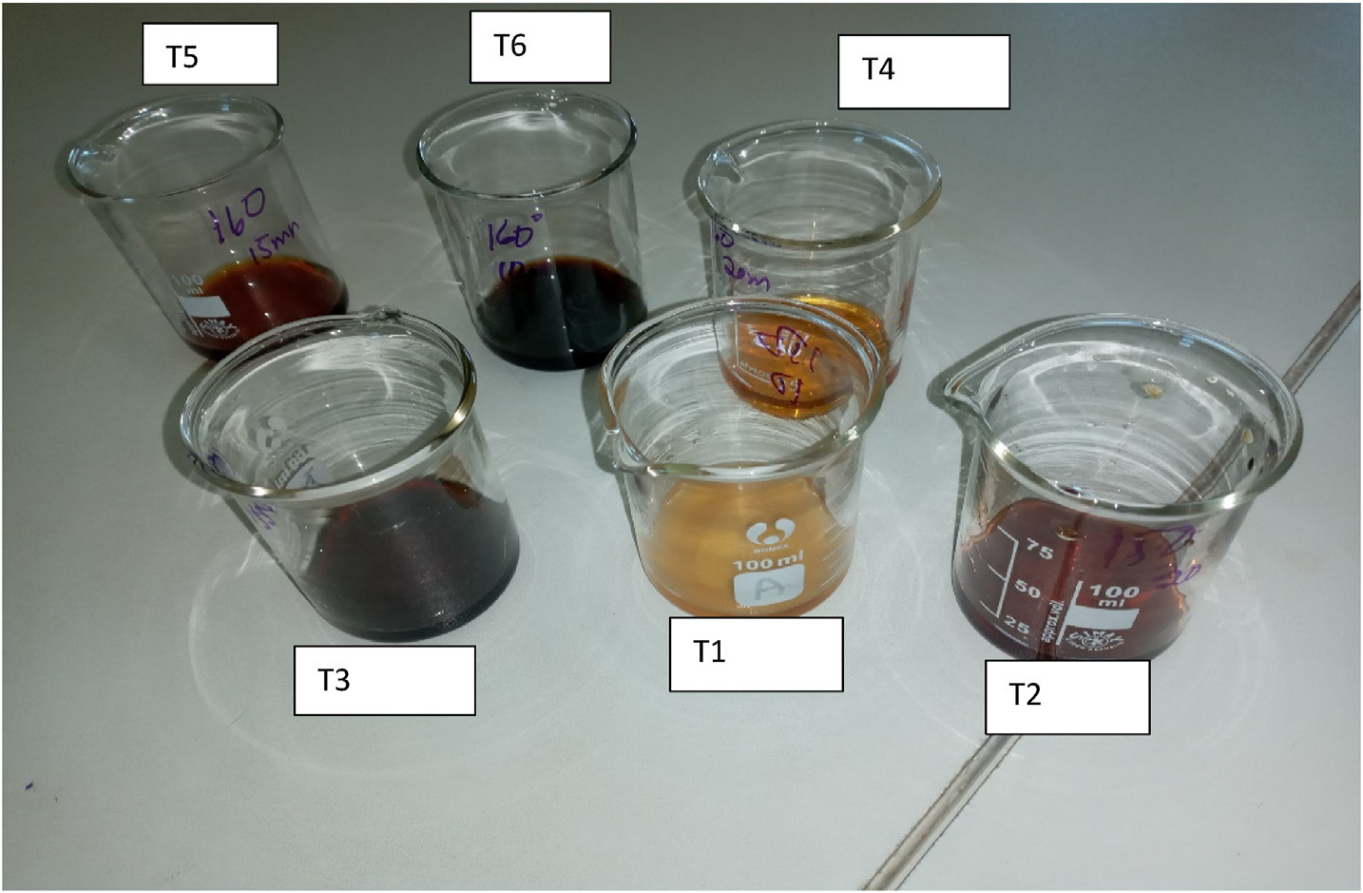
The brown colour developed for each treatment (T1 = treatment 1 at 150 °C for 10 min; T2 = treatment 2 at 150 °C for 15 min; T3 = treatment 3 at 150 °C for 20 min; T4 = treatment 4 at 160 °C for 10 min; T5 = treatment 5 at 160 °C for 15 min; T6 = treatment 6 at 160 °C for 20 min).

Browning index (BI) tended to increase with increase in roasting time both at 150 and 160 °C (Table 2). The browning index of Motlopi coffee at 160 °C was significantly (p < 0.05) higher than BI at 150 °C (Table 2). The lowest BI was observed for treatment 1 (150 °C for 10 min) and the highest BI was observed for treatment 6 (160 °C for 20 min) (Table 2). The results indicated that there is an increase in the browning index value with an increase in roasting time and temperature. The brown colour developed for each treatment is indicated in Figure 2. According to Illy and Viani (1999), browning index defined as brown colour purity, is one of the most common indicators of browning in sugar containing foods. Farsi et al. (2007) reported that the browning in roasted foods is mainly due to the development of non-enzymatic reactions such as Maillard reaction and sugar caramelization products. The authors further explained that the Maillard reaction predominates when components such as reducing sugars and amines which include amino acids, peptides and proteins react with each other during thermal treatments in food processing. As a result, thermally processed foods generally contain various levels of Maillard reaction products, which are the right time–temperature indicators for determining the extent of a thermal process. The development of brown pigments in coffee is enhanced by non-enzymatic browning and pyrolysis reactions that occur during the roasting process and this give the coffee a darker colour (Fikry et al., 2019). It was also reported that the reddish colour of coffee increases with increase in temperature and time of roasting resulting in darker colour coffee (Fikry et al., 2019).

The titratable acidity (TA) of Motlopi coffee decreased with an increase in roasting time both at 150 and 160 °C (Table 2). The TA of Motlopi coffee at 150 °C was significantly (p < 0.05) higher than TA at 160 °C (Table 2). The highest TA was observed for treatment 1 (150 °C for 10 min) and the lowest TA was observed for treatment 6 (160 °C for 20 min). The traditionally prepared Motlopi coffee had comparable TA with treatment 2 (150 °C for 15 min). According to Clifford, 1985a, Clifford, 1985b, the most abundant acid in coffee arabica and coffee robusta is chlorogenic acid. The four aliphatic acids namely formic, acetic, glycolic, and lactic acids formed during coffee roasting process constitute the major fraction of acidity in coffee (Cordoba et al., 2021). It was determined that increased roasting temperatures results in degradation of chlorogenic acid precursors and lower extractable total chlorogenic acid concentrations (Blumberg et al., 2010; Fuller and Rao, 2017). Bähre and Maier (1996) has demonstrated that TA shows better correlation to sourness than pH 4.0. Rao and Fuller (2018) found that the sourness of coffee correlates well with TA titrated to pH 6.041. Di Mattia et al. (2007) suggested that phenolic acids deprotonate at pH values greater than 8.42. The family of chlorogenic acid compounds are known to contribute significantly to the antioxidant activity of coffee. The polyphenolic compounds in coffee have an antioxidant and antiradical activity in radical-mediated mutagenic pathways (Somporn et al., 2011).

### 3.2 Consumer acceptability test of Motlopi coffee

The results for consumer acceptability test for the laboratory made and traditional Motlopi coffee are indicated in Table 3. The results showed that the laboratory made Motlopi coffee had significantly higher (p < 0.05) scores for taste and body than the traditional Motlopi coffee (Table 3). However, no significant difference (p > 0.05) was observed between the experimental and traditionally made Motlopi coffee for colour and aroma (Table 3). The laboratory made Motlopi coffee had significantly higher (p < 0.05) overall acceptability score than the traditionally made Motlopi coffee (Table 3). The results of this experiment showed that through manipulation of roasting temperature and roasting time, it is possible to prepare Motlopi coffee with improved sensory quality as compared to the traditionally prepared Motlopi coffee.

**Table 3:** Consumer acceptability test of laboratory made and traditionally prepared Motlopi coffee (n = 30).

Attributes	Treatment			
	Experimental	Traditional	P-value	
Colour	6.87 ± 1.07	6.83 ± 1.49	0.9195	
Taste	6.50^a^ ± 0.97	5.60^b^ ± 1.52	0.0147	
Aroma	6.23 ± 0.73	5.63 ± 1.52	0.0831	
Body	6.13^a^ ± 0.78	5.43^b^ ± 1.14	0.0069	
Overall acceptability	6.57^a^ ± 0.73	6.13^b^ ± 0.89	0.0300	

n = number of panelists; Means with different superscript letters in a row are significantly different (p < 0.05): Values in the Table are mean scores provided by the panelists for each attribute.

Colour is a crucial attribute that can be used as a quality control indicator during a roasting process. The highest colour score of the brew (6.87) was observed at a roasting temperature of 150 °C and roasting time of 15 min and the control had a lower score of 6.83 (Table 3). The results showed that there was no significant difference between the two scores. The most preferred colour of Motlopi coffee brew was observed for the experimental Motlopi coffee. The colour score increased as the roasting time and temperature increased (Ng et al., 2014). The change in colour is due to the browning index which resulted in Maillard reaction. In the present study, the intensity of the brown colour developed increased with increase in roasting time and roasting temperature as can be seen from the browning index experiment (Figure 2).

In terms of taste attributes, the highest score was 6.50 which was obtained at a roasting temperature of 150 °C and roasting time of 15 min (Table 3). The lowest score was obtained for the traditional Motlopi coffee with a score of 5.60. The result showed that the most preferred taste for the Motlopi coffee brew was that of the experimental Motlopi coffee. There was a significant difference in taste scores between the experimental Motlopi coffee and the traditional Motlopi coffee. According to Spiro and Selwood (1984), the changes in the taste for coffee brews is related to the change in acidity of coffee during the roasting process. As the roasting time and temperature increase, there is a decrease in the acidity of the coffee brew.

Aroma is also considered as an important quality indicator in coffee. Aroma is a chemical sense stimulated by the chemical properties of odour molecules which must reach the olfactory bulb to interact with the olfactory cells in the olfactory mucosa (Farah et al., 2009). The high aroma score of the coffee brew was observed for a roasting temperature of 150 °C and a roasting time of 15 min which was the experimental Motlopi coffee with a score of 6.23 and low score was observed for a traditionally prepared Motlopi coffee brew with a score of 5.63 (Table 3). In the case of arabica coffee and robusta coffee, the aroma of coffee is determined by a complex balance of the various volatile and non-volatile compounds formed during roasting of coffee (Hameed et al., 2018). The temperature and time of roasting determine the types of the aroma compounds formed during roasting of coffee (Hameed et al., 2018). The extent of aroma- and flavour-producing reactions were found to be reduced due to higher roasting temperature (over roasting) (Hameed et al., 2018). Thus, optimum roasting temperature is required to produce the desired aroma compounds in coffee. The higher aroma score observed in the experimental Motlopi coffee as compared to the traditionally prepared Motlopi coffee in the present study could be related to the difference in the roasting condition (temperature and time) between the two processes.

Coffee’s body describes the physical properties and tactile sensations perceived by the mouth such as the sense of heaviness or mouthfeel as the coffee settles on the tongue (Gloess et al., 2013). The result showed that the highest score of the body for coffee brew (6.13) was observed at a roasting temperature of 150 °C and roasting time of 15 min and the lowest score (5.43) was obtained for the traditional Motlopi coffee. The most preferred body of the Motlopi coffee was observed for the experimental coffee. There was a significant difference in body scores between the two coffee types. According to Anisa et al. (2017), coffee body contributes to a sensation of the coffee’s richness including its aroma and taste.

The highest overall acceptability score of the Motlopi coffee brew was obtained for the experimental Motlopi coffee prepared at a roasting temperature of 150 °C and a roasting time of 15 min with a score of 6.57. The results showed that the experimental Motlopi coffee had higher overall acceptability as compared to the traditionally prepared Motlopi coffee with a significantly higher taste and body scores.

In the case of arabica coffee and robusta coffee, postharvest processing conditions significantly influence the organoleptic quality of the final coffee (Hameed et al., 2018). Postharvest processing conditions in particular roasting, grinding and brewing/extraction influence the majority (60%) of the quality attributes of coffee (Hameed et al., 2018). The quality of the final coffee is significantly influenced by the various biochemical reactions (Maillard reactions, Strecker degradation, caramelization, pyrolysis) that occur during roasting which in turn determine the production of over 1000 different types of aroma compounds (Hameed et al., 2018). A range of flavour and colour imparting compounds are also generated during roasting (Hameed et al., 2018).

Grinding is another very important step in coffee processing, as size reduction, particle distribution, and uniformity determine the final quality attributes of coffee (Hameed et al., 2018). The fineness/grind size of the particles determine the extractability of soluble solids, acids, and aroma compounds in coffee, and this eventually contribute to the body of the beverage (Hameed et al., 2018). Grinding enhances the degree of interaction between water and coffee particles due to increase in surface area of the particles (Hameed et al., 2018). Optimal grinding grade permits contact of the maximum surface area with the hot water to attain a high-quality coffee brew (Severini et al., 2015; Hameed et al., 2018).

Moreover, the sensory quality of coffee is also influenced by extraction/brewing techniques such as coffee/water ratio, extraction temperature and extraction time (Hameed et al., 2018). For example, acidity is more pronounced at lower coffee brew temperature (Gloess et al., 2013; Cordoba et al., 2021). The difference in the overall acceptability (liking) observed between the experimental Motlopi coffee and the traditional Motlopi coffee in the current study could be attributed to differences in the degree of roasting and extent of grinding applied during the production of the two coffee types.

Some panelists who participated in the sensory panel commented that the Motlopi coffee powder especially of the traditional Motlopi coffee was coarse and it would have been better if very fine powders were used. Some commented that the traditional Motlopi coffee powder was not uniformly roasted and this was reflected in the taste and aroma of the coffee where the traditional Motlopi coffee had burnt flavour and smoky aroma as opposed to the experimental Motlopi coffee. They indicated that charred powder particles were visible in the traditionally prepared Motlopi coffee and this could be the reason for the dark brown colour of the traditional Motlopi coffee. The comments made by the panelists also confirmed that the degree of roasting and the extent of grinding of the Motlopi coffee powder played a major role in the observed difference in the overall acceptability between the experimental and traditional Motlopi coffees.

## 4 Conclusion

Coffee-like brew prepared from roasted Motlopi roots is an important beverage that is widely consumed by communities in Botswana. The results showed that roasting temperature and roasting time have major influence on the sensory acceptability of Motlopi coffee. Roasting condition also affected the physicochemical properties of Motlopi coffee powder, which have influence on the final brewed coffee. The experimental coffee had better overall quality as compared to the traditional Motlopi coffee. This suggests the possibility of making Motlopi coffee with good physicochemical properties and higher consumer acceptability through determination of optimal processing conditions especially roasting temperature/time and particle size of the ground coffee.

In the current study, analysis of the major chemical components related to the quality of Motlopi coffee and descriptive sensory analysis were not conducted on Motlopi coffee. Thus, there is a need for analysis of the major chemical composition linking to the quality of Motlopi coffee and consideration of descriptive sensory analysis on Motlopi coffee.

## Declarations

### Author contribution statement

Kenanao Otsogile: Conceived and designed the experiments; Performed the experiments; Analyzed and interpreted the data; Wrote the paper.

Eyassu Seifu: Conceived and designed the experiments; Analyzed and interpreted the data; Wrote the paper.

Geremew Bultosa: Conceived and designed the experiments; Analyzed and interpreted the data; Wrote the paper.

### Funding statement

This work was supported by the Department of Tertiary Education Financing of Botswana.

### Data availability statement

Data included in article/supp. material/referenced in article.

### Declaration of interest’s statement

The authors declare no conflict of interest.

### Additional information

No additional information is available for this paper.
